# Animal models of chronic experimental asthma — strategies for the identification of new therapeutic targets

**DOI:** 10.1186/1745-6673-3-S1-S4

**Published:** 2008-02-27

**Authors:** Michael Wegmann

**Affiliations:** 1 Department of Clinical Chemistry and Molecular Diagnostics, Biomedical Research Center (BMFZ), Hospital of the Philipps-University, 35043 Marburg, Germany

## Abstract

Over the last decade mouse models of experimental asthma proved to be a valuable tool for the investigation of mechanisms that underlie acute allergic airway inflammation and development of airway hyperresponsiveness, two of the hallmarks of human asthma. Nevertheless, these acute models fail to reflect the aspects of this chronic disease because they do not represent any signs of chronicity and airway remodelling as it is defined by subepithelial fibrosis, goblet cell hyperplasia and airway smooth muscle cell hypertrophy. Recent mouse models were successful in overcoming these limitations by using chronic allergen-challenges. These new models of chronic experimental asthma now proved as a novel tool to examine the complex interaction of infiltrating inflammatory cells and structural cells such as fibroblasts and smooth muscle cells that ultimately leads to airway remodelling and stable airflow limitation. Recent studies clearly demonstrated that T helper 2 (TH2) cells and their typical cytokines play a critical role not only in airway inflammation but also in the development of airway remodelling. Since the transcription factor GATA-3 is essential for TH2 cell development and the production of several TH2 type cytokines this intracellular molecule represents a new promising target for therapeutic intervention in asthma that might even effect airway remodelling.

## Overview

Allergic bronchial asthma is a chronic inflammatory disease of the airways that is characterized by allergic airway inflammation and development of airway hyperresponsiveness (AHR). Another hallmark of asthma involves various structural changes of the airway wall such as airway fibrosis and smooth muscle cell hyperplasia summarized as airway remodelling [1]. Over the last 15 years animal models, especially mouse models, of experimental asthma have been intensively used to investigate the immuno-pathological mechanisms that ultimately lead to the formation of this disease. Mostly, these models utilized systemic sensitization to foreign proteins such as ovalbumin (OVA) adsorbed to the adjuvant aluminium hydroxide (alum) to induce a T-helper-2 (TH2) cell triggered immune-response together with marked production of OVA-specific IgE. Once sensitized, these animals developed airway eosinophilia and AHR after inhalation or intra-nasal application of OVA aerosols or OVA-solutions [2]. Such models provided insight into the immunological mechanisms of acute allergic inflammation, helped to identify key cytokines such as interleukin 4 (IL-4), IL-5 and IL-13 that play critical roles in the regulation of the local inflammatory process [3] and proofed valuable to investigate the neuro-immunological dysregulation underlying the development of AHR [4].

## Mouse models of chronic experimental asthma

However, since allergic airway inflammation in these models is induced by short term exposure to the aerosolized allergen and, therefore, does not persist for longer than 10 days, these models are limited regarding the modelling of the chronic aspects of asthma, more precisely airway remodelling and its patho-physiological consequences that might result in stable airflow limitation. Furthermore, pathological aspects of experimental asthma in these acute models are reduced to the central airways. Since asthma, allergic inflammation and airway remodelling is observed in peripheral airways of asthmatic patients as well [5,6] and structural changes especially of distal airways play a critical role in the development of irreversible airflow limitation [7] these aspects represent important hallmarks for disease modelling in animals.

Over the last few years airway remodelling became a field of special interest in asthma research since it is largely resistant to medication [8] and is an important factor to develop irreversible airflow limitation, resulting in progress of asthma severity [9]. To overcome these limitations recently many advanced models have been established. Most of these models use systemic sensitization to ovalbumin (OVA) adsorbed to aluminium hydroxide (alum) followed by repeated exposure to aerosolized OVA for 4-8 weeks [10-13]. Within these models it is interesting to note that similar results are achieved with a huge variety of OVA-aerosol concentrations (0.1 – 2.5%) as well as the frequency of allergen challenges (2–7 times a week).

However, the modelling of these chronic aspects of asthma in animals requires clear definition of airway remodelling. The pathological analysis on bronchial specimens of asthmatic patients revealed goblet cell hyperplasia of the airway epithelium, hyperplasia of the submucosal glands, thickening of the epithelial basement membrane, extracellular matrix deposition in the subepithelial layer and hypertrophy and hyperplasia of the bronchial smooth muscle cells [14]. To reflect these hallmarks of airway remodelling, we sensitized BALB/c mice to OVA by three intra-peritoneal (i.p.) injection of OVA adsorbed to alum followed by a twelve week long OVA challenge period with two challenges a week on consecutive days [15]. These animals developed chronic allergic inflammation that in contrast to other models affected the entire bronchial tree and even persisted after six weeks of OVA challenge discontinuation. This chronic airway inflammation was further associated with persistent AHR and stable airflow limitation as indicated by reduction of baseline midexpiratory airflow (EF50). In contrast to other models of chronic experimental asthma, proximal as well as distal airways revealed marked deposition of collagen fibres in the airway wall especially in the lamina propria. The airway wall was additionally thickened by appearance of myofibrocytes and enlargement of the airway smooth muscle layer indicating airway smooth muscle hypertrophy. Goblet cell hyperplasia could be observed in all sections of the bronchial tree. Since this model reflects human airway remodelling to a high degree it could be useful for the investigation of the mechanisms that underlie this special issue of human asthma.

## Lymphocytes and eosinophils in airway remodelling

The actual concept of asthma pathogenesis begins with the differentiation of naïve allergen-specific T-helper cells towards TH2 cells that are characterized by the expression of the transcription factor GATA-3 and production of several typical cytokines such as IL-5 that controls the activity and the survival of eosinophils. These cells represent the main effector cell type of the allergic airway inflammation and produce a variety of cytotoxic mediators and cationic proteins such as major basic protein (MBP) and eosinophil cationic protein (ECP) that ultimately lead to epithelial damage [16]. This further triggers several mechanisms focussing on the reconstitution of the epithelial integrity. One of the main mediators involved in this process is transforming growth factor-beta (TGF-β) that is produced by eosinophils and structural cells such as bronchial epithelial cells and smooth muscle cells [17]. This cytokine on the one hand possesses anti-inflammatory properties that help to limit the inflammatory process in the airways. On the other hand it triggers airway remodelling since it induces pro-collagen production in fibroblasts and transformation of these cells into smooth muscle actin containing myofibroblasts [18,19].

We utilized our model of chronic experimental asthma to assess the effect of a novel therapeutic strategy that targets eosinophils on airway remodelling and lung function. Since the infiltration of these cells into the airways depends on interaction of the C-C chemokine receptor-3 (CCR-3) and its ligands from the eotaxin family, we antagonized this chemokine receptor by using a low molecular weight compound [20]. To mimic the human situation as closely as possible treatment with the CCR-3 antagonist started not until allergic airway inflammation has already been established, but no signs of airway remodelling could be observed. Systemic application of 30mg/kg body weight reduced the infiltration of eosinophils into the broncho-alveolar lumen and into the airway tissue by about 70%, whereas infiltration of other inflammatory cells was not effected significantly. Additionally, neither subepithelial fibrosis nor hyperplasia of smooth muscle cells or goblet cells could be observed in animals treated with the CCR-3 antagonist. Although airway eosinophilia could not be reduced entirely airway reactivity to metacholine was nearly normalized in these animals indicating that eosinophils play an important, but not an exclusive role in the processes leading to airway remodelling and AHR. Both, involvement of eosinophils in development of AHR and in development of airway remodelling remain issues of special interest and are discussed highly controversial. In models of acute experimental asthma development of AHR is usually accompanied by airway eosinophilia. Nevertheless, a substantial number of studies clearly demonstrate that factors other than airway eosinophilia are essential for the development of AHR. These factors involve TH2-type cytokines such as IL-4 and IL-13 as well as signalling via the Fc-epsilon receptor [21-23]. Furthermore, Tournoy *et al*. report about a model of acute experimental asthma where airway eosinophilia and development of AHR seem to be disconnected [24]. Similar results were achieved by Siegle *et al* in a mouse model of chronic experimental asthma [25]. The discussion about eosinophils and development of AHR came to a climax when the groups of Humbles and Lee reported about the results of their studies in the same issue of the *science* magazine. Humbles *et al.* used the Δdbl GATA mouse which is completely deficient for the eosinophil lineage due to deletion of a high affinity GATA site of the GATA-1 promotor in a mouse model of experimental asthma to demonstrate that eosinophils are not obligatory for allergen-induced lung dysfunction [26]. In contrast, Lee utilized the transgenic PHIL mouse that is specifically devoid of eosinophils, to show that these are required for development of AHR [27].

Even though both studies seem to be contradictory regarding the role of eosinophils within the development of AHR, both studies linked eosinophils to the development of airway remodelling. Whereas Lee *et al*. reported the lack of goblet cell metaplasia in PHIL mice Humbles *et al*. clearly demonstrated that eosinophil deficient animals were protected from airway fibrosis and smooth muscle cell hyperplasia. These findings receive further support from studies that report about the profibrotic effects of eosinophils on fibroblasts *in vitro*[28-30] and the results concerning antagonization of CCR-3 that were produced by Fulkerson *et al*. [31] and by our group.

Besides the fact that TH2 cells induce goblet cell hyperplasia and increased mucus production in asthmatic patients by secreting IL-13 [32], very little is known about the contribution of T cells to airway remodelling. Few recent studies focussed on this special issue and presented data that in fact suggest the participation of TH2 cells in the development of airway fibrosis and smooth muscle hyperplasia. Komai *et al*. investigated the effect of a neutralizing anti-CD4 antibody in a mouse model of chronic experimental asthma [33]. The i.p. administration of this antibody during the chronic OVA aerosol challenge did not only result in diminished airway inflammation and reduced amounts of TH2-type cytokines in broncho-alveolar lavage fluids, but also decreased goblet cell hyperplasia and airway fibrosis. This was further accompanied by the observation that neutralizing CD4 downsized the level of TGF-β in BAL fluids as well. Additional experiments from the same group focussed on the question whether typical TH2-type cytokines such as IL-4 and IL-5 may contribute to airway remodelling. For this purpose IL-4 deficient animals were sensitized to OVA and chronically exposed to an OVA aerosol. Compared to wild type animals, IL-4 deficient animals did not develop allergic airway inflammation and did not reveal any signs of airway remodelling as indicated by lack of goblet cell hyperplasia and subepithelial fibrosis. Similar results could be observed in animals that were deficient for the alpha chain of the IL-5 receptor. In contrast, OVA-sensitized IL-5 transgenic (tg) mice that systemically overexpress IL-5 showed even higher levels of TGF-β and enhanced subepithelial fibrosis than wild type animals after chronic OVA aerosol challenge [13].

Although all these studies reported about reduced airway remodelling after abolishing CD4 positive T cells or TH2-type cytokines one could argue that all these effects might be indirect since these lowered effects were always associated with diminished airway eosinophilia. However, a study by Ramos-Barbon gave evidence that TH2 cells can directly induce airway remodelling [34]. In contrast to all previously mentioned studies these experiments were carried out in the rat, since this species is from an anatomical point of view more suitable to mimic changes of the airway smooth muscle layer. In this study CD4 positive T cells from OVA-sensitized rats, that were challenged chronically with OVA aerosol and revealed signs of airway remodelling including airway smooth muscle cell (ASMC) hyperplasia, were adoptively transferred to naïve rats. After OVA aerosol challenge these CD4 positive T cells infiltrated the airway tissue and could be localized in close contact to muscle cells of the airways and airway vessels. This was further associated with increased airway smooth muscle mass indicating ASMC hypertrophy. By using immuno-histological staining methods Ramos-Barbon *et al*. were able to clearly demonstrate that ASMCs from adoptively transfered animals exhibited increased proliferation and reduced apoptosis. All the data presented in this study support the idea that airway smooth muscle remodelling, as an important feature of asthma, is driven by antigen-specific T helper cells that require direct myocyte-T cell contact.

## Conclusion

Animal models of experimental asthma have elucidated the central role of TH2 cells and their typical cytokines in the acute inflammatory process resulting in the formation of asthma. To answer the question whether these cells are further involved in pathophysiological processes ultimately leading to airway remodelling and chronification of the disease improved models of chronic experimental asthma that are able to reflect the chronic aspects of this disease as close as possible had to be established. Recent studies used such models to demonstrate that TH2 cells play a critical role in asthma chronification especially in airway remodelling as well. Therefore, these cells still represent promising target cells for therapeutic intervention in asthmatic patients. After embryonic development the pleiotropic transcription factor GATA-3 remains to be expressed in bone marrow derived cells such as eosinophils, basophils, mast cells and especially in T cells [35,36]. During T helper cell development GATA-3 expression promotes TH2 responses through at least three different mechanisms and therefore represents a crucial event in T cell polarization [37-40]. Furthermore, overexpression of GATA-3 leads to increased airway remodelling in a mouse model of chronic experimental asthma [41]. So due to its central role within the pathology of asthma GATA-3 represents an interesting target for new anti-allergic strategies, that might even be able to interfere with processes that ultimately lead to airway remodelling.

## Competing interests

The author declares that he has no competing interests.
